# Haemophilus influenzae Bacteremia With Polyarthritis and Uterine Fibroids in a 31-Year-Old Woman: A Case Report

**DOI:** 10.7759/cureus.102318

**Published:** 2026-01-26

**Authors:** Lila Dudley, FNU Varnika, Nyla Naeem, Bryan Dawkins, Nergess Taheri

**Affiliations:** 1 Medicine, Nova Southeastern University Dr. Kiran C. Patel College of Osteopathic Medicine, Davie, USA; 2 Family Medicine, Lakeside Medical Center, Belle Glade, USA

**Keywords:** bacteremia of unknown source, friable cervix, giant uterine fibroid, gram-negative bacteremia, haemophilus influenzae, ovarian cyst, polyarticular pain

## Abstract

*Haemophilus influenzae* (*H. influenzae*)* *can, in rare cases, infect the female reproductive tract, typically presenting with fever, lower abdominal pain, and vaginal discharge. Rarely, infection manifests with diffuse polyarticular pain and systemic symptoms. We report the case of a woman in her early 30s who presented to the emergency department (ED) with a two-day history of polyarticular joint pain, malaise, chills, and weakness. Examination revealed tachycardia and tenderness of multiple joints on passive and active range of motion. Laboratory evaluation showed leukocytosis, elevated C-reactive protein (CRP), elevated troponin, iron-deficiency anemia, and a positive urinalysis. Despite persistent polyarticular pain, the patient developed worsening lower abdominal pain, chest pain, and progressive lower extremity weakness. Blood cultures grew *H. influenzae*, confirming bacteremia. Imaging revealed a large intramural fibroid causing mass effect and a right ovarian cyst. Pelvic examination demonstrated a friable, elongated cervix, though vaginal fluid culture and Gram stain were negative. This case highlights an atypical presentation of *H. influenzae* bacteremia with an unclear primary source, emphasizing the diagnostic challenges posed by extrapulmonary manifestations of this organism.

## Introduction

*Haemophilus influenzae* (*H. influenzae*) is a Gram-negative bacterium whose strains are classified based on whether the organism is encapsulated (typable) or unencapsulated (non-typable) [1]. Since the introduction of the *H. influenzae* type b (Hib) conjugate vaccine, non-typable *H. influenzae* (NTHi) is the most common cause of invasive disease in the United States, averaging 1.3 cases per 100,000 population annually [1]. The most common presentations of invasive *H. influenzae* in the United States are pneumonia, meningitis, and occult bacteremia [1]. Most commonly, invasive *H. influenzae* affects preterm neonates, older patients (>65 years old), immunocompromised patients, and pregnant and postpartum women.

In healthy individuals, *H. influenzae* most commonly presents as pneumonia, as it colonizes the nasopharyngeal mucosa and can be aspirated into the lower respiratory tract, leading to pulmonary infection [2]. In recent years, however, *H. influenzae* has been increasingly recognized as a cause of early-onset neonatal sepsis and severe upper genital tract infections in pregnant women [3]. When the pathogen colonizes the genital mucosa and normal anatomical barriers are disrupted perinatally, it can ascend to infect the newborn or upper female reproductive organs [3]. Reported cases of *H. influenzae*-associated upper female genital tract infections are most commonly linked to the disruption of anatomical barriers and are rare outside the context of pregnancy, instrumentation, or intrauterine device (IUD) placement [4]. When *H. influenzae* infects the female reproductive tract, the common presenting symptoms are fever, lower abdominal pain, and discharge.

## Case presentation

A woman in her early 30s presented to the emergency department (ED) with a two-day history of diffuse polyarticular pain, malaise, chills, and weakness. The pain involved multiple joints, including the wrists, elbows, sternum, hips, lower back, and knees, with the left knee being most affected. She attributed the severity of left knee pain to a motor vehicle accident one month prior. She denied headaches, dizziness, rashes, urinary or abdominal symptoms, recent illness, sick contacts, sexual activity, travel, trauma, or lifestyle changes. Her past medical history included menorrhagia, untreated human papillomavirus (HPV) infection (last Pap smear unknown), prediabetes, last menstrual period one month prior, and a childhood hernia repair.

On physical examination, the wrists, knees, elbows, and hips were diffusely tender during passive and active motion but not to palpation alone. There was no joint edema, erythema, or warmth. The remainder of the examination was unremarkable.

**Figure 1:**
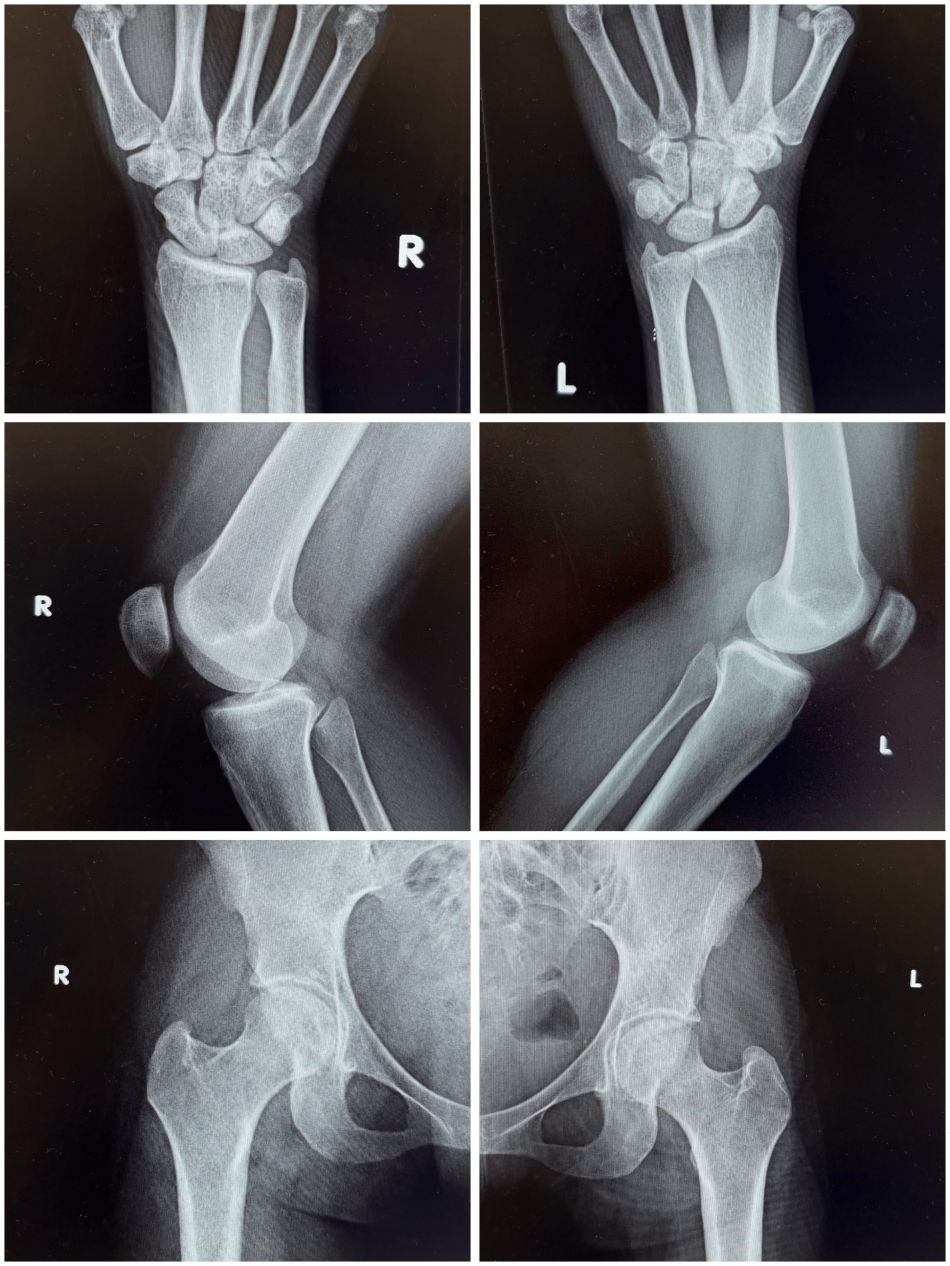
Radiographs of the bilateral wrists, knees, and joints on the day of admission, showing clear joint spaces and well-defined bone structures. There is no evidence of joint space narrowing, effusions, or osteopenia, supporting a noninfectious etiology of the joint pain.

Initial emergency department workup included meeting systemic inflammatory response syndrome (SIRS) criteria with tachycardia (98 beats per minute {bpm}) and elevated white blood cells (WBC) (22.5 × 109/L). Sepsis criteria were then met based on a positive urinalysis (1+ ketones, 1+ protein, 2+ leukocyte esterase, 5-10 WBC, 1+ bacteria, and 0-2 granular casts) (Table 1), despite the patient being asymptomatic for urinary complaints. Laboratory findings included elevated C-reactive protein (CRP) (24.3 mg/dL), elevated troponin (64.7 ng/L, delta score negative), and iron-deficiency anemia (Table 2). Infectious screening was negative for sickle cell disease, hepatitis B/C, HIV, gonorrhea, chlamydia, and syphilis. Radiographs of the hips, knees, lumbar spine, and wrists showed no osseous abnormalities, joint space changes, or effusion (Figure 1). She was admitted for sepsis secondary to presumed asymptomatic urinary tract infection and further evaluation of polyarticular pain.

**Table 1:** Urinalysis results on the day of admission. WBC, white blood cells; HPF, high-powered field

Test	Patient’s Value	Reference Range
Ketones	1+	Negative
Protein	1+	Negative to trace
Leukocyte esterase	2+	Negative
Bacteria	1+	None seen
WBC	5-10	0-5/HPF
Granular casts	0-2	None seen

**Table 2:** Laboratory results on the day of admission. BUN, blood urea nitrogen; CK, creatine kinase; WBC, white blood cell; MCV, mean corpuscular volume

Test	Patient’s Value	Reference Range
Glucose	85	70-110 mg/dL
BUN	21	7-18 mg/dL
Creatinine	0.87	0.50-1.20 mg/dL
Potassium	3.4	3.5-5.1 mmol/L
Alkaline phosphatase	128	46-116 U/L
C-reactive protein	24.3	<5 mg/L
Lactic acid	1.1	0.4-1.9 mmol/L
CK	88	26-308 U/L
Troponin	64.7	0.0-51.4 ng/L
WBC	22.5	4.8-10.8 × 10⁹/L
Hemoglobin	7.8	12.7-14.7 g/dL
Hematocrit	25.1	37.9%-43.9%
MCV	74.9	80.0-94.0 fL
Iron	5	35-150 µg/dL

During the first two days of hospitalization, blood cultures grew Gram-negative rods, while urine cultures were negative. She remained afebrile with WBC down-trending to 14.5 × 10^9^/L. Despite this, she developed worsening symptoms: minor bilateral wrist swelling, progressive lower extremity weakness, diffuse chest pain described as “pressure-like,” and severe lower abdominal pain radiating to the hips and back. Repeat examinations revealed moderate to severe groin tenderness and mild lower abdominal tenderness. Pain was managed with IV morphine, and empiric antibiotic therapy with IV cefepime and metronidazole was initiated to provide broad-spectrum coverage of Gram-positive, Gram-negative, aerobic, and anaerobic organisms, while the source of infection remained unclear.

**Figure 2:**
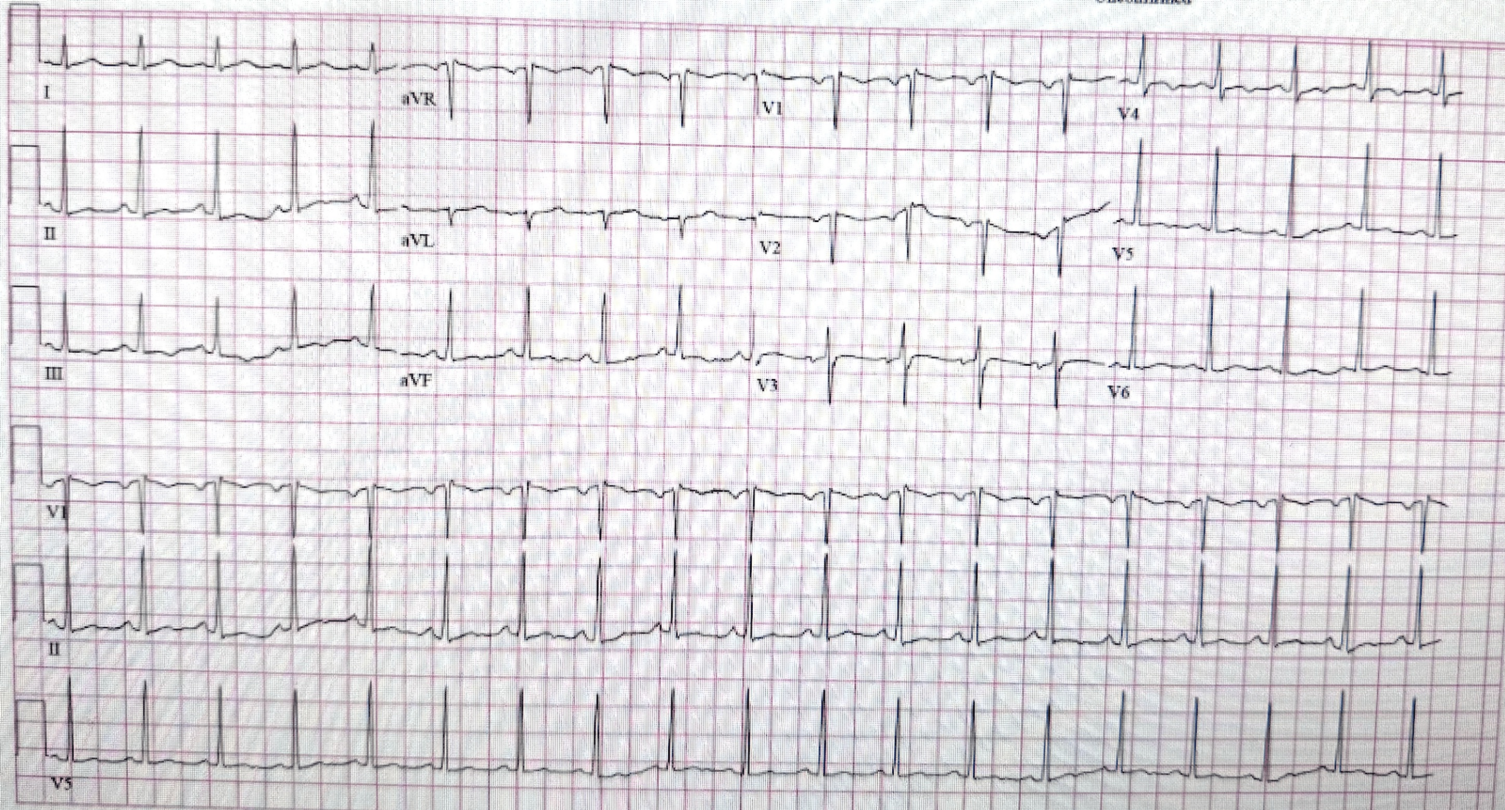
EKG displaying no pathological findings, supporting the notion that the patient’s chest pain was not cardiac in origin. EKG: electrocardiogram

**Figure 3:**
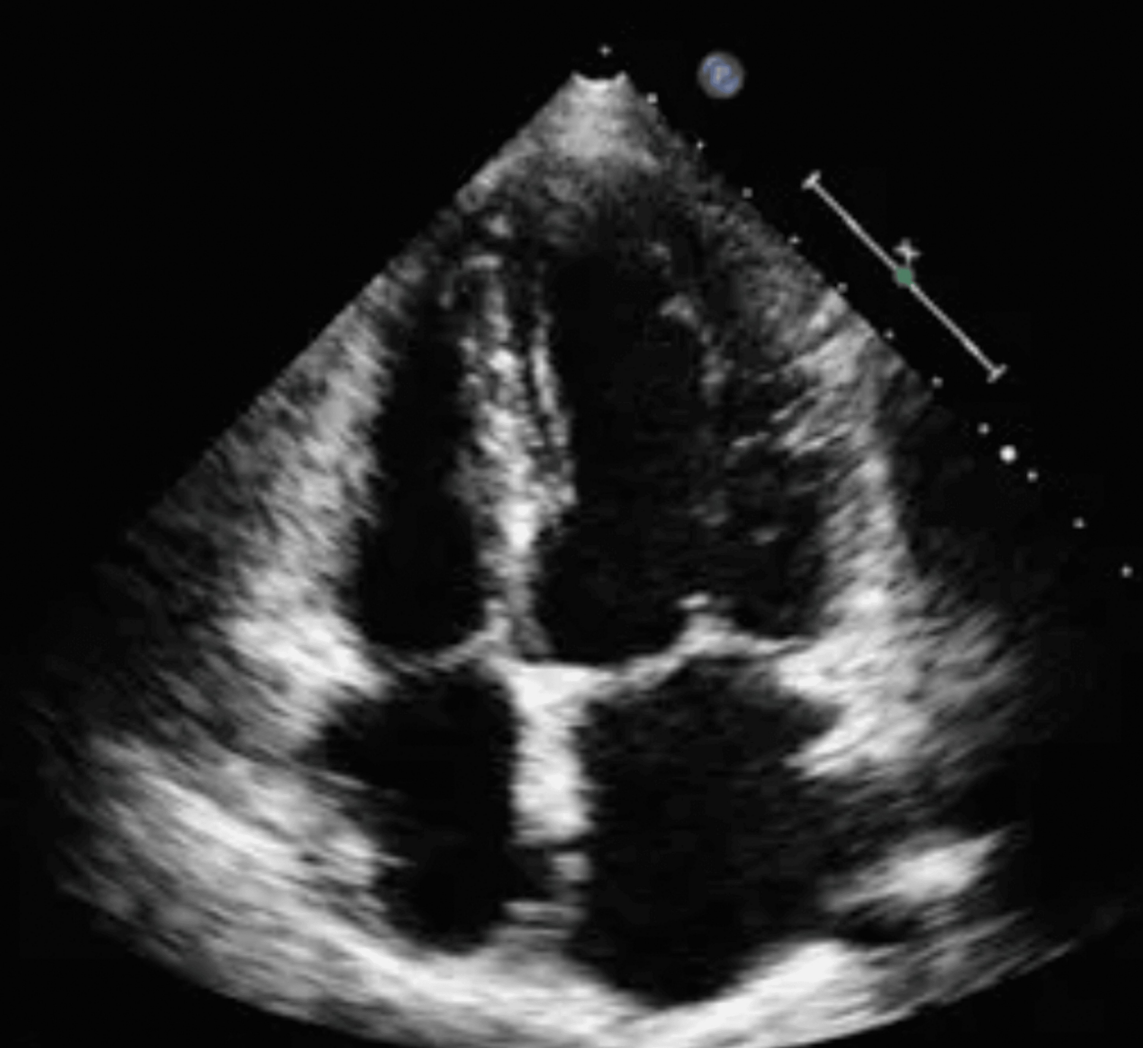
Echocardiogram displaying no pathological findings, including no vegetations or pericardial effusions, supporting the notion that the source of infection is not cardiac.

**Figure 4:**
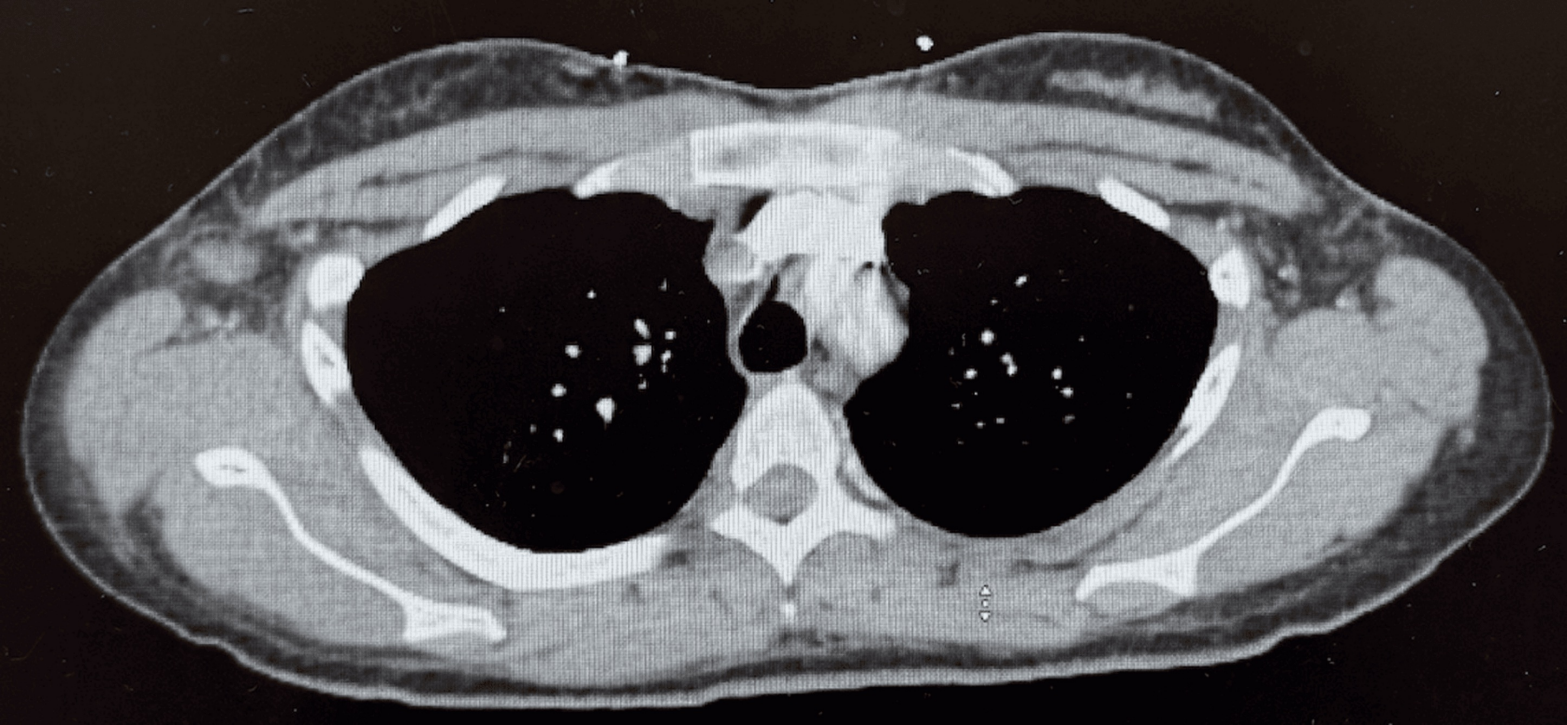
CT of the chest displaying no pathological findings, including consolidations, pleural effusions, interstitial infiltrates, or abscesses, supporting the notion that the source of infection is not pulmonary. CT: computed tomography

**Figure 5:**
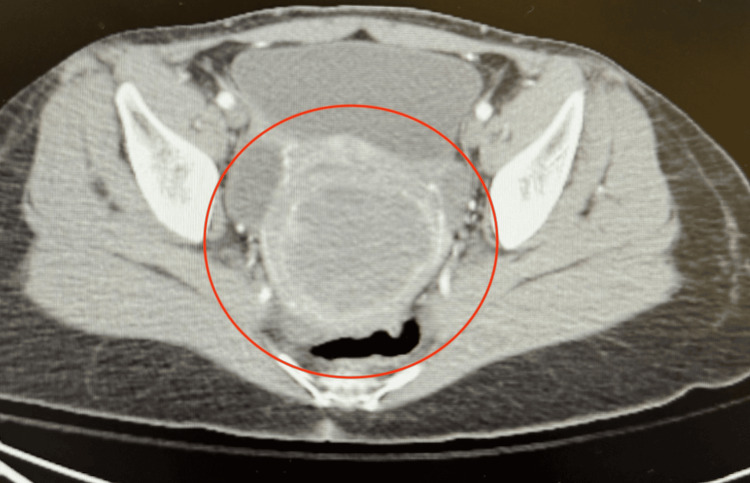
CT of the pelvis revealing a uterine lesion suggestive of a fibroid or cystic change. CT: computed tomography

**Figure 6:**
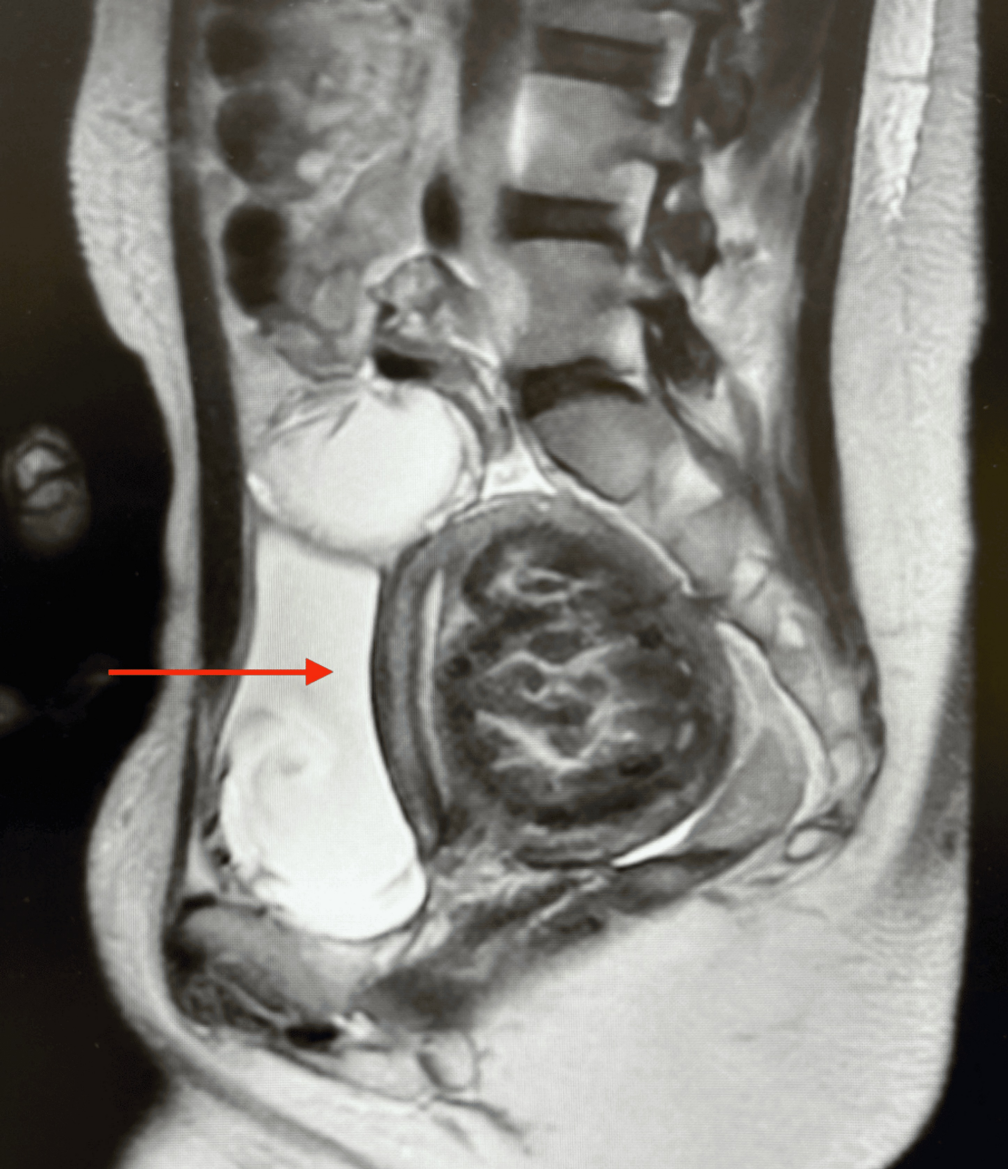
MRI of the pelvis displaying enlarged fibroid uterus with a 7 cm mass causing endometrial displacement.

Further workup included cardiopulmonary evaluation (electrocardiogram {EKG}, chest computed tomography {CT}, echocardiogram, respiratory panel, and repeat troponins) (Figure 2-4), which was unremarkable. Abdominal and pelvic imaging revealed a uterine lesion suggestive of a fibroid with cystic changes (Figure 5). Pelvic ultrasound confirmed an enlarged uterus with a single intramural fibroid (6.2 × 6.4 × 6.7 cm) and a right ovarian cyst (4.0 × 1.9 × 3.9 cm). Despite these findings and her abdominal pain, she had no vaginal discharge, history of severe dysmenorrhea, sexual activity, or gynecologic instrumentation to suggest a reproductive tract infection. An MRI of the lumbar spine and pelvis was then ordered, and it showed a mild degenerative disc bulge at L5-S1 without stenosis or evidence of abscess/osteomyelitis. An MRI of the pelvis revealed an enlarged fibroid uterus with a 7 cm mass causing endometrial displacement, a thickened cervix possibly related to malignancy, free pelvic fluid, and a 4.6 cm right ovarian cyst (Figure 6).

On day 4 of admission, blood cultures finally grew *Haemophilus influenzae*. Antibiotics were adjusted to include oral doxycycline for gynecologic-related complications, alongside the existing treatment of IV cefepime and metronidazole. Due to concern for gynecologic infection per the infectious disease team, a pelvic examination was advised. The patient consented to a pelvic examination, which revealed a long, friable cervix with an abnormal collapsing os. There was equivocal tenderness in the inguinal and groin regions. Genital cultures and ThinPrep cytology were obtained. Cultures came back negative, Gram stain grew no organisms, and cytology revealed no cervical intraepithelial neoplasia (CIN), but HPV co-testing was positive.

Due to services being unavailable, the patient was transferred to an outside facility with obstetrics and gynecology for further evaluation of uterine findings. During her stay, she continued IV ceftriaxone, oral doxycycline, and metronidazole, along with pain management. Repeat blood cultures at five days were negative.

## Discussion

This case of *Haemophilus influenzae* bacteremia presented with diffuse polyarticular pain and uterine abnormalities without a definitive infectious source. This raises important questions as to whether the uterine findings represent the primary source of bacteremia and, if so, whether they adequately explain the patient’s clinical presentation. Additionally, given that polyarticular pain was the presenting symptom, the possibility of the joints as the primary source of infection must also be considered.

*Haemophilus influenzae* has been documented to infect the female reproductive tract, causing conditions such as acute endometritis, vaginitis, urethritis, uterine abscesses, and incomplete septic abortions [4-6]. Typically, these infections present with lower abdominal pain, vaginal discharge, and fever, along with physical findings of adnexal or cervical motion tenderness. Most reported cases involve risk factors such as intrauterine device (IUD) placement, gynecologic instrumentation, or postpartum status [7].

Several case reports illustrate this association. One patient with *H. influenzae* bacteremia with a history of IUD placement was diagnosed with acute endometritis after presenting with abdominal pain, fever, and vaginal discharge. Imaging revealed leiomyomas, and endometrial biopsy confirmed neutrophilic infiltration and polymerase chain reaction (PCR) positivity for *H. influenzae*, despite negative Gram stain and culture [7]. Another case of *H. influenzae* bacteremia in a patient with lower abdominal pain described an intra-myometrial abscess as the source of the symptoms. Following abscess rupture and hysterectomy, *H. influenzae* was confirmed as the cause of the abscess and subsequent *H. influenzae* bacteremia [4]. A third report identified micro-abscesses within adenomyoma tissue in a patient with bacteremia and positive vaginal cultures, despite imaging showing no abscess [5].

In contrast to the cases discussed, our patient lacked the hallmark features of pelvic infection. She remained afebrile throughout hospitalization, had no vaginal discharge, and only developed abdominal pain on the second day of admission. Cervical cultures and Gram stains were negative. Unlike prior cases, uterine samples were unable to be obtained due to resource limitations, leaving the presence of *H. influenzae* in the uterus unconfirmed. However, given that *H. influenzae* endometritis and micro-abscesses have been reported despite negative imaging and genital Gram stain, uterine involvement cannot be excluded.

Abscess formation in uterine fibroids occurs following the infarction of the mass with subsequent bacterial seeding [8]. As the leiomyoma outgrows its blood supply or thrombosis occurs, tissue degeneration and necrosis create an environment conducive to bacterial colonization [8]. The infection of the neoplasm may occur due to hematogenous seeding or due to ascending infection, establishing the possibility that even if *H. influenzae* was confirmed within the uterus, the fibroid itself may represent a secondary site of infection rather than the definitive primary source.

An alternative consideration is whether the joints were the primary source of infection, given that polyarticular pain was the presenting symptom. One case reported by Kenzaka et al. described a similar presentation of *H. influenzae* bacteremia in a patient with polyarticular pain but with diffuse joint swelling [9]. Upon joint aspiration, the authors discovered cultures positive with *H. influenzae*, but they could not conclude whether the joint was the source of infection or a secondary site seeded by bacteremia [9].

If the joints were the source of the infection, reactive arthritis and septic arthritis should be considered, given that they are both causes of joint pain attributed to infection. Reactive arthritis presents with asymmetric oligoarthritis involving large lower extremity joints and is associated with ocular, skin, and cardiac manifestations [10]. According to the diagnostic criteria, however, a key requirement for the diagnosis of reactive arthritis is a preceding symptomatic infection, most commonly urethritis or enteritis, which our patient never had [10].

Septic arthritis presents with monoarticular or oligoarticular joint pain, marked joint swelling, fever, and elevated inflammatory markers [11]. Radiographs do not typically show signs of septic arthritis; therefore, definitive diagnosis requires synovial fluid analysis [11]. However, in this case, the absence of significant joint swelling made joint aspiration unwarranted, alongside the fact that our patient lacked significant risks for septic arthritis such as skin infection, intra-articular injections, prosthetic joints, diabetes mellitus, and immunosuppression [11].

Objectively, it is not possible to rule septic arthritis as the cause of the bacteremia, given that synovial cultures were never obtained. However, it must be considered that another risk factor for developing septic arthritis is sepsis [11]. Therefore, even with a positive synovial culture, we would be unable to determine whether joint involvement was a primary or secondary source, just as in the case described by Kenzaka et al. [9].

Ultimately, without direct sampling of uterine tissue or synovial fluid, the source of bacteremia remains uncertain. Moreover, even if sampling from the uterus or joints was performed, *H. influenzae* occupying either location would not confirm it as the source of primary infection, given that both may result from hematogenous seeding.

## Conclusions

We report a rare case of *Haemophilus influenzae* bacteremia without an identifiable primary source, presenting with diffuse polyarticular pain in the absence of overt joint inflammation. Diagnostic evaluation was notable only for *H. influenzae* bacteremia and a large uterine fibroid, while common sources of infection, including urinary, cardiac, and pulmonary etiologies, were excluded. Although the uterine abnormality and joints were considered potential sources, the absence of characteristic risk factors, imaging findings, and confirmatory tissue or synovial sampling precluded definitive localization. This case underscores the diagnostic challenges posed by bacteremia of unclear origin and highlights the potential for hematogenous seeding to produce atypical clinical manifestations, emphasizing the importance of maintaining a broad differential diagnosis in similar presentations.
